# Tumor cell invasion in blood vessels assessed by immunohistochemistry is related to decreased survival in patients with bladder cancer treated with radical cystectomy

**DOI:** 10.1186/s13000-021-01171-7

**Published:** 2021-11-22

**Authors:** Birgitte Carlsen, Tor Audun Klingen, Bettina Kulle Andreassen, Erik Skaaheim Haug

**Affiliations:** 1 Department of Pathology, Vestfold Hospital Trust, Halfdan Wilhelmsens allé 17, N-3103 Tonsberg, Norway; 2 Department of Research, Cancer Registry of Norway, Ullernchausseen 64, N-0379 Oslo, Norway; 3 Department of Urology, Vestfold Hospital Trust, Halfdan Wilhelmsens allé 17, N-3103 Tonsberg, Norway

**Keywords:** Bladder cancer, Blood and lymph vessel invasion, CD31, D2–40

## Abstract

**Background:**

Lymphovascular invasion (VI) is an established prognostic marker for many cancers including bladder cancer. There is a paucity of data regarding whether the prognostic significance of lymphatic invasion (LVI) differs from blood vessel invasion (BVI). The aim was to examine LVI and BVI separately using immunohistochemistry (IHC), and investigate their associations with clinicopathological characteristics and prognosis. A secondary aim was to compare the use of IHC with assessing VI on standard HAS (hematoxylin-azophloxine-saffron) sections without IHC.

**Methods:**

A retrospective, population –based series of 292 invasive bladder cancers treated with radical cystectomy (RC) with curative intent at Vestfold Hospital Trust, Norway were reviewed. Traditional histopathological markers and VI based on HAS sections were recorded. Dual staining using D2–40/CD31 antibodies was performed on one selected tumor block for each case.

**Results:**

The frequency of LVI and BVI was 32 and 28%, respectively. BVI was associated with features such as higher pathological stages, positive regional lymph nodes, bladder neck involvement and metastatic disease whereas LVI showed weaker or no associations. Both BVI and LVI independently predicted regional lymph node metastases, LVI being the slightly stronger factor. BVI, not LVI predicted higher pathological stages. BVI showed reduced recurrence free (RFS) and disease specific (DSS) survival in uni-and multivariable analyses, whereas LVI did not. On HAS sections, VI was found in 31% of the cases. By IHC, 51% were positive, corresponding to a 64% increased sensitivity in detecting VI. VI assessed without IHC was significantly associated with RFS and DSS in univariable but not multivariable analysis.

**Conclusions:**

Our findings indicate that BVI is strongly associated with more aggressive tumor features. BVI was an independent prognostic factor in contrast to LVI. Furthermore, IHC increases VI sensitivity compared to HAS.

**Supplementary Information:**

The online version contains supplementary material available at 10.1186/s13000-021-01171-7.

## Background

In bladder cancer (BC), the detection of vascular invasion (VI) by tumor tissue in RC specimens has been shown to be associated with adverse outcomes [1–3]. The migration of tumor cells into vascular channels is considered an early step of metastatic spread. Since Lotan et al. [4] reported that lymphovascular invasion was associated with poor prognosis in BC, several studies have confirmed this observation, showing that the presence of tumor cells within vascular spaces is a predictor of nodal metastasis, recurrence and cancer specific death [5–13]. However, most of these studies did not discriminate between tumor cells in blood vessels and lymphatic vasculature, and the feature of invasion is often reported as `lymphovascular`. It has been suggested that malignant tumors appear to have different preferences regarding lymphatic or hematogenous spread [14], a feature that may be important for further disease progress. D2–40 has shown to be a highly sensitive and specific marker for the detection of endothelium in lymphatic vessels [15]. For the detection of endothelial cells in blood vessels, CD31 is frequently used, as it is commonly regarded as the most sensitive and specific marker of endothelial differentiation [16], however, this marker is not completely specific for blood vessel endothelium as it may also show weak reactivity in lymphatic endothelium. Interestingly, Afonso et al. [17] compared H&E (hematoxylin-eosin) assessment with IHC (CD31, D2–40), separating single tumor cell invasion and tumor emboli, and found that tumor emboli in CD31 positive vessels remained an independent prognostic factor on multivariable analysis of overall survival (OS) in a series of 83 cases of infiltrating carcinoma. In a study of immunohistochemically assessed LVI and BVI in primary pT1 urothelial carcinomas including 32 patients, Gakis et al. [18] observed a positive predictive value of LVI and/or BVI for predicting stage≥pT2a disease at RC of 100%. However, the study had low power with only 16 patients showing ≥pT2 at cystectomy, and did not include survival analyses. If vascular and lymphatic invasion share the same prognostic traits remains nevertheless unclear.

The aim of this study was to examine the prevalence and prognostic significance of BVI and LVI assessed by IHC, and their associations with respect to subsequent metastatic disease and survival, in a population- based series of invasive bladder tumors (stage pT1–4) in RC specimens.

## Materials and methods

### Study population

We studied a consecutive, population-based series treated with RC at Vestfold Hospital Trust. Since 2013, the hospital has had a multi-regional center function for cystectomy in patients with BC, covering regions in South-Eastern Norway comprising approximately 20% of the Norwegian population with around 1,060,000 residents.

This series accounted for 292 patients with invasive BC diagnosed from transurethral resection for bladder tumor (TURBT) treated with RC with curative intent between 2000 and 2018. Patients with distant metastases at the time of diagnosis, no residual malignant tissue in the cystectomy specimen (ypT0), concomitant ureter cancer or urachal adenocarcinomas were excluded.

Regarding primary treatment, 48 patients (16%) received neoadjuvant chemotherapy and 24 patients (8%) received adjuvant chemotherapy. We did not have any records on palliative radiation or chemotherapy during follow up.

The median follow up period was 48 months (range 19–180). None of the patients was lost to follow up due to insufficient information.

### Clinical variables

The clinical and follow up data were collected from the Urological Register at Vestfold Hospital Trust, and medical records at Vestfold Hospital Trust and referring hospitals with respect to disease recurrences, survival time, date for last status recorded (date, month and year) and deaths.

The cause of death was recorded based on evaluation of medical certificates of death obtained from medical journals, and in a few cases on telephone calls to general practitioners and nursing home doctors. In a very few cases autopsy had been performed. Occurrence of any relapse and/or metastatic spread during the course of disease was recorded with date and site based on evaluation of CT scans reported in the medical journals. Only in a very few cases the metastases were histologically verified by biopsy. For 44 patients the follow up information was obtained from a questionnaire sent by postal service.

### Pathological assessment

All original histological sections were reviewed by one pathologist (BC). Traditional prognostic histopathological parameters such as tumor type according to WHO 1973/ISUP 2004 [19], stage according to TNM 2017 classification [20], concomitant carcinoma in situ, lymph node status, tumor subsites, status of surgical resection margins and VI based on HAS were recorded, as well as any divergent tumor differentiation according to the WHO 2016 criteria [19]. We did not record histological grade as the vast majority of invasive bladder cancers now are considered high grade [21, 22].

As for divergent differentiation within the urothelial carcinoma category, the cases were divided into four groups. The groups were subsequently fused into two; micropapillary and other variants. We did not record the percentage of divergent histology within the tumors. The presence of VI based on HAS sections was evaluated according to the morphological criteria defined by Algaba [23] as a free floating tumor embolus with tightly cohesive clusters of cells with smooth borders, within an unequivocal endothelium-lined space, preferentially with the minimum of two endothelial cells per space. No attempt was made to separate the vessel types based on HAS sections, nor to distinguish vessels based on size.

### Immunohistochemistry

Tissue specimens were fixed in 10% buffered formalin and embedded in paraffin. All original sections were reviewed, and one representative block from the bladder wall with tumor tissue including deepest invasive focus and adjacent peritumorous tissue, if any, was selected for further staining. In all cases, the selected block was equal to a block with observed VI.

Immunohistochemical staining was performed on Benchmark Ultra (Ventana, Roche) on standard 4–5 μm sections, after overnight baking at 60 °C. Dewaxing and antigen retrieval was preserved in the instrument (fully automated, “on board”). Dual staining with Podoplanin, clone D2–40 (M3619, Dako, Agilent) for lymphatic vessel staining and CD31 (clone JC70A, M0823, Dako, Agilent) for blood vessel staining was performed. Briefly, the sections were pretreated with ULTRA Cell conditioning 1 (ULTRA CC1) for 36 min at 95 °C and then incubated with D2–40, diluted 1:25, for 32 min at 37 °C. Staining for D2–40 was carried out using UltraView DAB (ref. no.760–500). As for CD31, the sections were incubated for 32 min at 37 °C, diluted 1:40. Staining was carried out using UltraView Red (ref. no. 760–501).

Finally, the sections were counterstained with hematoxylin for 1 min (Shandon Instant Hematoxylin Kit, ThermoScientific).

**Fig. 1:**
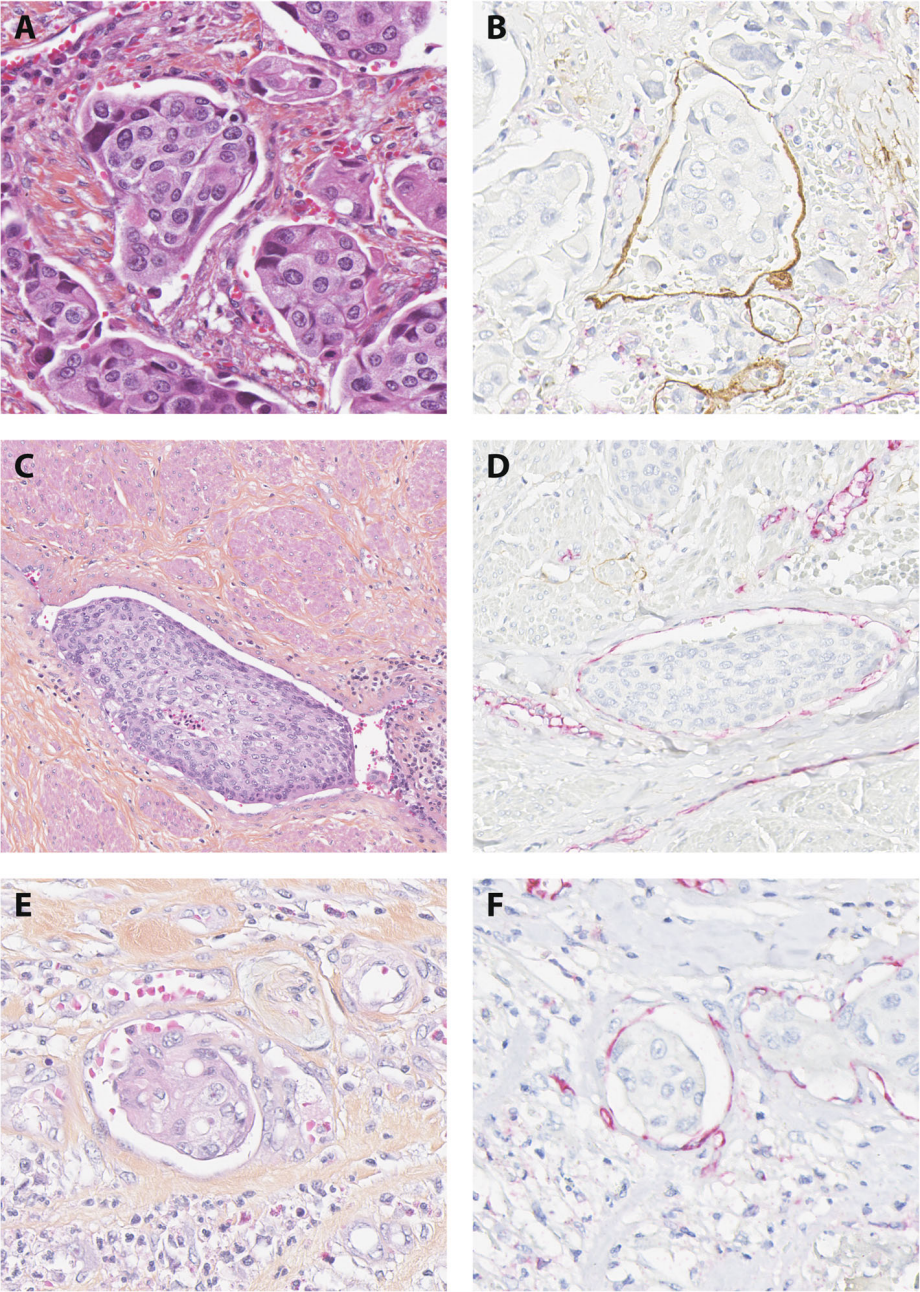
Histological images of LVI and BVI by immunohistochemistry (× 400). Footnotes: **A**, VI, HAS section. **B**, the same vessel, positive for D2–40 (LVI). **C**, VI, HAS section. **D**, the same vessel, positive for CD31 (BVI). **E**, VI, HAS section. **F**, the same vessel, positive for CD31 (BVI)

All stained sections were examined by a pathologist (BC), and the observer was blinded for clinicopathological information. A few cases difficult to interpret were consulted with another experienced pathologist (TAK), and consensus was reached. We recorded LVI to be present if tumor tissue, i.e. single tumor cells, clusters of tumor cells or tumor thrombi were located within one or more than one D2–40 positive structure with weak or negative CD31 staining. BVI was reported when single tumor cells, clusters of tumor cells or tumor thrombi were detected in one or more than one CD31-positive and D2–40 negative vessel (Fig. 1).

2 × 25 cases were arbitrarily chosen and reviewed by two pathologists for assessing Kappa values (Cohen’s kappa coefficient) for interobserver reliability. As for the assessment of VI based on HAS sections, the Kappa value was 0.6, whereas BVI and LVI based on IHC showed very good agreement, with Kappa values of 0.9 and 1.0, respectively.

### Statistical analyses

All statistical analyses were performed by using IBM SPSS Statistics, V. 26,0 (IBM, Armonk, USA). 292 patients were available for analyses. Associations between different categorical variables were assessed by Pearson’s χ^2^ test. We considered both recurrence free survival (RFS) and time to death from bladder cancer (disease specific survival, DSS). The date for cystectomy was used as the entry point. Patients who died from other causes were censored at time of death. Unadjusted/observed survival was presented using Kaplan Meier curves and a log-rank test for evaluating differences in survival curves. The proportional hazard assumption for VI, together with standard clinicopathological variables was further analysed by log-log plots. We also investigated interactions between variables that were significantly associated with survival in the Cox regression analysis. A multivariable analysis was conducted for BVI and LVI adjusting for standard prognostic variables. Multivariable logistic regression was applied to assess the ability of various clinicopathological variables to predict regional lymph node metastases and higher pathological stages. A result was considered significant when *p* < 0.05.

### Ethics

Study participants submitted broad, informed consent, and data were de-identified for the analyses. The study was approved by the Regional Ethics Committee of South Eastern Norway (REK 2019/532).

## Results

### Patient characteristics

Table 1 presents the patient characteristics of the study cohort, in total and stratified by gender; 68 (23%) were female, 224 (77%) were male. The median age at cystectomy was 72 years. In 256 patients (88%), regional lymph node dissection (LND) had been performed, of which 86 (34%) had lymph node metastases. Urothelial carcinomas comprised 274 (94%) of cases. Positive surgical margins were observed in 29 specimens (10%).

**Table 1:** Patient characteristics for the study cohort, in total and stratified by gender

	Total (n,%)	Female (n,%)	Male (n,%)
	292	68 (23)	224 (77)
**Age**			
40–68	103 (35)	24 (36)	79 (35)
69–76	95 (33)	20 (29)	75 (34)
77–88	94 (32)	24 (35)	70 (31)
**Female**	68 (23)		
**Male**	224 (77)		
**Pathological stage**			
pT1	39 (13)	6 (8.8)	33 (15)
pT2a	46 (16)	9 (13)	37 (17)
pT2b	27 (9,2)	5 (7.4)	22 (9.8)
pT3a	63 (23)	13 (19)	50 (22)
pT3b	83 (28)	25 (37)	58 (26)
pT4a	33 (11)	10 (15)	23 (10)
pT4b	1 (0.3)	0	1 (0.4)
**Performed lymph node dissection**	256 (88)	57 (84)	199 (89)
N0	170 (66)	41 (72)	129 (65)
N1–3	86 (34)	16 (28)	70 (35)
**No lymph nodes dissected, median, IQR**	17 (11–17-22)	16 (11–16-22)	17 (11–17-22)
**Surgical resection margin status**			
Positive surgical margins	29 (10)	6 (8.8)	23 (10)
CIS in urethral and/or ureteric margins	8 (2.7)	3 (4.4)	5 (2.2)
**Histologic tumor type**			
Urothelial carcinoma	274 (94)	61 (90)	213 (95)
Squamous cell carcinoma	11 (3.8)	5 (7.4)	6 (2.7)
Adenocarcinoma	2 (0.7)	1 (1.5)	1 (0.4)
Small cell neuroendocrine carcinoma	5 (1.7)	1 (1.5)	4 (0.8)
**Divergent differentiation**	113 (41)	31 (51)	82 (39)
Squamous	61 (22)	17 (28)	44 (21)
Micropapillary	23 (8.4)	4 6.6)	19 (8.9)
Glandular	6 (2.2)	1 (1.6)	5 (2.3)
Others/mixed	23 (8.4)	9 (15)	14 (6.6)
**Concomitant carcinoma in situ**	136 (47)	25 (37)	111 (50)
**Tumor subsites**			
Trigone ± ureteric orifices	32 (11)	9 (13)	23 (10)
Bladder neck	49 (17)	7 (10)	42 (19)
Diverticula	12 (4.1)	0	12 (5.4)
Other	199 (68)	52 (77)	147 (66)
**Chemotherapy**			
Neoadjuvant	47 (16)	11 (16)	36 (16)
Adjuvant	24 (8.2)	4 (5.9)	20 (8.9)

Out of 292 patients, we observed relapse of disease in 142 (49%), of which 56 events were local recurrences, 33 regional lymph node metastases, 42 non-regional lymph node metastases and 140 various distant metastases. Among the 166 patients (57%) who died during the follow up period, 132 (80%) died from bladder cancer and 34 (20%) died from other causes.

### Lymphovascular invasion assessed with and without IHC

Based on HAS sections, VI was found in 31% (*n* = 91) of cases and was significantly associated with histopathological variables such as higher pathologic stages (OR 5.8, CI 3.0–11.1, *p* < 0.001), regional lymph node metastases (OR 5.2, CI 2.9–9.2, *p* < 0.001), micropapillary variant histology (OR 2.8, CI 1.2–6.8, *p* = 0.019), bladder neck involvement (OR 3.4, CI 1.8–6.5, *p* < 0.001), positive surgical margins (OR 3.6, CI 1.6–7.9, *p* = 0.001), local recurrence (OR 2.1, CI 1.2–3.7, *p* = 0.006) and distant metastases (OR 3.2, CI 1.9–5.5, *p* < 0.001) (Table 2). By IHC, 150 (51%) patients were positive. Regarding treatment, LVI was associated with patient subgroup treated with adjuvant chemotherapy (OR 2.4, CI 1.0–5.5, *p* = 0.042). As for BVI and VI assessed by HAS, no associations were found regarding neither neoadjuvant nor adjuvant treatment.

**Table 2:** Associations between different categories of vascular invasion with and without IHC and various clinicopathological variables

	BVI				LVI				VI			
Variables	Neg (n,%)	Pos (n,%)	OR (95% CI)	***p*** value	Neg (n,%)	Pos (n,%)	OR (95% CI)	***p*** value	Neg (n,%)	Pos (n,%)	OR (95% CI)	***p*** value
**No. Patients (*n*** **= 292)**	211 (72)	81 (28)			200 (69)	92 (32)			201 (69)	91 (31)		
**Age at surgery, median**												
40–72	120 (57)	34 (42)	1		107 (54)	47 (51)	1		112 (56)	42 (46)	1	
73–88	91 (43)	47 (58)	1.8 (1.1–3.1)	0.022	93 (46)	45 (49)	1.1 (0.7–1.8)	0.70	89 (44)	49 (54)	1.5 (0.8–2.4)	0.13
**Female**	50 (24)	18 (22)			45 (23)	23 (25)	1		49 (24)	19 (21)	1	
**Males**	161 (76)	63 (78)	0.9 (0.5–1.7)	0.79	155 (77)	69 (75)	1.1 (0.6–2.0)	0.64	152 (76)	72 (79)	0.8 (0.5–1.5)	0.51
**Pathological stage**												
t1 + 2	101 (48)	11 (14)	1		83 (42)	29 (32)	1		99 (49)	13 (14)	1	
t3 + 4	110 (52)	70 (86)	5.8 (2.9–11.7)	< 0.001	117 (58)	63 (68)	1.5 (0.9–2.6)	0.10	102 (51)	78 (86)	5.8 (3.0–11.1)	< 0.001
**Nodal status (*n*** **= 256)**												
N0	140 (74)	30 (45)	1		130 (73)	40 (51)			138 (78)	32 (41)	1	
N1–3	49 (26)	37 (55)	3.5 (1.9–6.3)	< 0.001	48 (27)	38 (49)	2.6 (1.5–4.5)	0.001	39 (22)	47 (60)	5.2 (2.9–9.2)	< 0.001
**Diverg diff (*n*** **= 274)**												
Other (ref. gr. UTC, no diverg diff)	123 (89)	38 (86)	1		115 (91)	46 (81)	1		116 (91)	45 (79)	1	
Micropapillary	16 (7.4)	6 (14)	1.2 (0.4–3.3)	0.71	11 (9)	11 (19)	2.5 (1.0–6.2)	0.042	11 (8.7)	12 (21)	2.8 (1.2–6.8)	0.019
**Tumor subsites**												
Other	189 (90)	54 (67)	1		171 (86)	72 (78)	1		179 (89)	64 (70)	1	
Bladder neck	22 (10)	27 (33)	4.3 (2.3–8.1)	< 0.001	29 (14)	20 (22)	1.6 (0.9–3.1)	0.12	22 (11)	27 (30)	3.4 (1.8–6.5)	< 0.001
**Surgical margin positivity**												
No	194 (92)	69 (85)	1		184 (92)	79 (86)	1		189 (94)	74 (81)	1	
Yes	17 (8)	12 (15)	1.9 (0.9–4.4)	0.084	16 (8)	13 (14)	1.9 (0.9–4.1)	0.10	12 (6)	17 (19)	3.6 (1.6–7.9)	0.001
**Perioperative chemotherapy**												
No	158 (75)	63 (78)	1		151 (76)	70 (76)			154 (77)	67 (74)	1	
Yes	53 (25)	18 (22)	0.9 (0.5–1.6)	0.61	49 (24)	22 (24)	0.9 (0.5–1.7)	0.91	47 (23)	24 (26)	1.2 (0.7–2.1)	0.58
Local recurrence*												
No	162 (77)	52 (64)	1		148 (74)	66 (72)	1		157 (78)	57 (63)	1	
Yes	49 (23)	29 (36)	1.8 (1.1–3.2)	0.030	52 (26)	26 (28)	1.1 (0.6–1.9)	0.685	44 (22)	34 (37)	2.1 (1.2–3.7)	0.006
**Distant metastasis**												
No	124 (59)	26 (32)	1		108 (54)	42 (46)	1		121 (60)	29 (32)	1	
Yes	87 (41)	55 (68)	3.0 (1.8–5.2)	< 0.001	92 (39)	50 (54)	1.4 (0.9–2.3)	0.19	80 (40)	62 (68)	3.2 (1.9–5.5)	< 0.001

*P* values were obtained using Pearson’s χ^2^ test. Abbreviations: *BV*I blood vessel invasion, *LVI* lymph vessel invasion, *VI* vascular invasion assessed without IHC. * recurrences in soft tissue at the surgical site and/or regional lymph nodes

Using IHC, 142 patients (49%) did not show any VI. 69 patients (24%) showed LVI only, 58 (20%) showed BVI only whereas 23 (8%) showed both LVI and BVI, giving overall frequencies of 32% (*n* = 92) and 28% (*n* = 81), respectively. Of the 201 patients (69%) classified on HAS sections to be VI negative, IHC classified 48 to have LVI and 25 to have BVI, indicating that 22% (*n* = 66) of cases previously reported to be negative showed VI by IHC. Of the 91 cases (31%) classified as VI positive on HAS sections, IHC could not confirm this in 6 cases (7%).

BVI and LVI were not significantly associated (OR 0.8, CI 0.5–1.4, *p* = 0.48). (Supplementary Table 1, additional file 1).

Table 2 shows the associations between clinicopathological variables and different categories of vascular invasion assessed with and without IHC. BVI was associated with higher age at cystectomy (OR 1.8, CI 1.1–3.1, *p* = 0.022), higher pathological stages (OR 5.8, CI 2.9–11.7, *p* < 0.001), positive lymph nodes (OR 3.5, CI 1.9–6.3, *p* < 0.001), bladder neck involvement (OR 4.3, CI 2.3–8.1, *p* < 0.001), local recurrence (OR 1.8, CI 1.1–3.2, *p* = 0.030) and distant metastases (OR 3.0, CI 1.8–5.2, *p* < 0.001). LVI was associated with positive lymph nodes (OR 2.6, CI 1.5–4.5, *p* < 0.001) and micropapillary divergent differentiation (OR 2.5, CI 1.0–6.2, *p* = 0.042).

VI assessed with and without IHC were significantly associated for both BVI and LVI, BVI showing the strongest association (OR 11.3, CI 6.2–20.4, *p* < 0.001, and OR 2.9, CI 1.8–5.0, *p* < 0.001, respectively). (Supplementary Table 2, additional file 1).

### The associations between BVI and LVI with regional lymph node metastases and stage

When including BVI and LVI in a multivariable logistic regression model adjusted for relevant clinicopathological variables, (gender, age, pathological stage, micropapillary divergent differentiation, neoadjuvant chemotherapy) both BVI and LVI independently predicted regional lymph node metastases (OR 2.7, CI 1.4–5.2, *p* = 0.003 for LVI and OR 2.4. CI 1.2–4.8, *p* = 0.018 for BVI) (Supplementary Table 3, additional file 1).

Including BVI and LVI in a multivariable logistic regression model adjusted for variables mentioned above, BVI was an independent predictor of higher pathological stages (OR 3.9, CI 1.8–8.8, *p* = 0.001). In contrast, the association for LVI was not significant (OR 1.5, CI 0.8–2.9, *p* = 0.23) (Supplementary Table 4, additional file 1). There was no interaction between BVI and LVI (regional lymph node metastases; *p* = 0.38. pathological stage; *p* = 0.70), and the impact of BVI and LVI did not change when exclusively including either of them in the model.

### Survival analysis

#### BVI and LVI by IHC

**Fig. 2:**
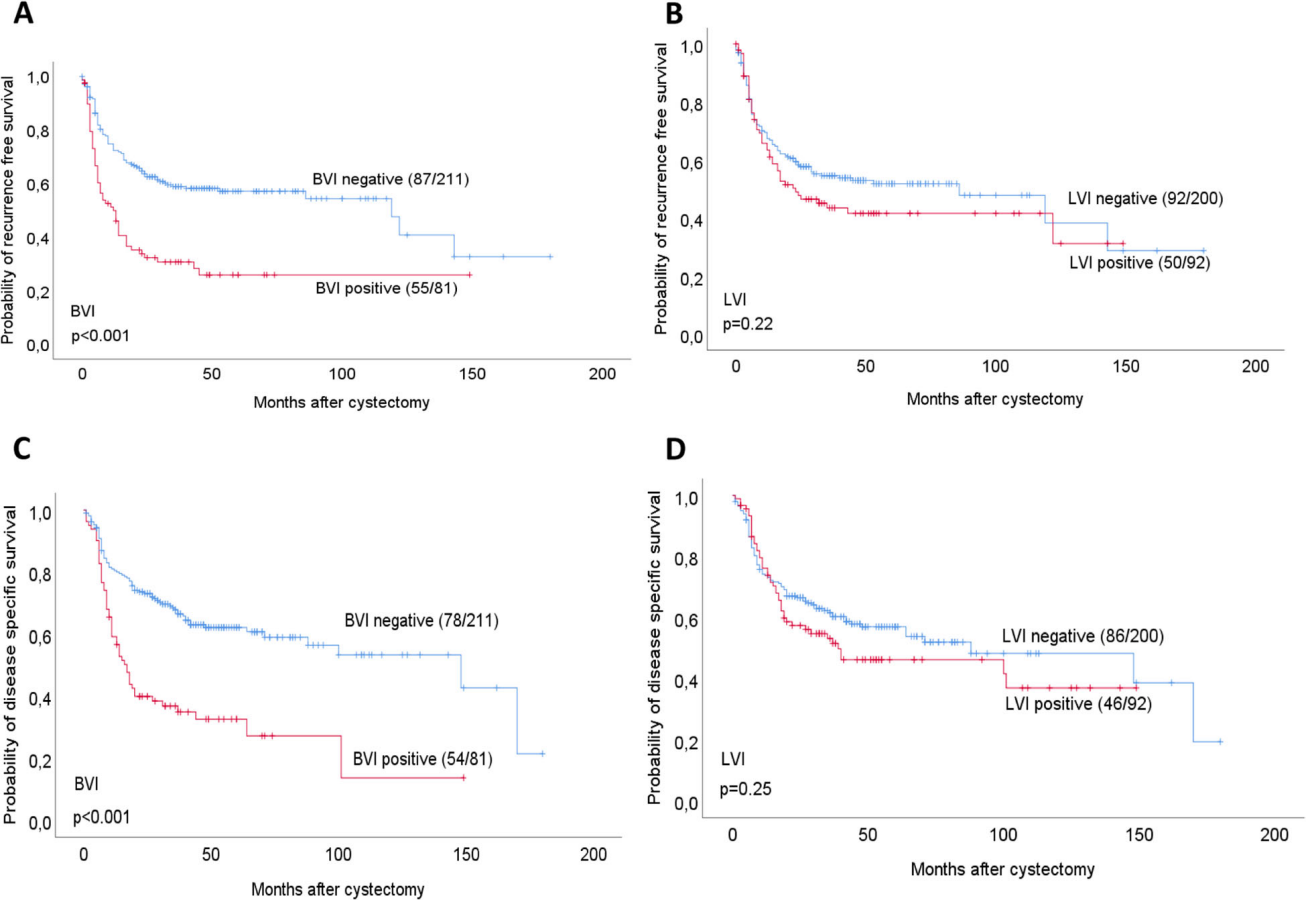
Recurrence free and disease specific survival related to BVI (**A**, **C**) and LVI (**B**, **D**). Footnotes: Survival curves are estimated by the Kaplan Meier method using the log-rank test for differences between subgroups. For each category, number of events/total number of cases are given

The observed survival is presented in Kaplan Meier curves (Fig. 2). BVI was significantly associated with reduced RFS and DSS (*p* < 0.001 for both). In contrast, LVI was not significantly associated with survival. When adjusting for relevant basic clinicopathological markers such as gender, age, pathological stage, lymph node status, surgical margin positivity and perioperative chemotherapy, BVI but not LVI was significantly associated with shorter RFS and DSS, as was also lymph node metastases, higher pathological stages and positive surgical margins (Table 3). These results were independent of perioperative chemotherapy.

**Table 3:** Multivariable analyses of RFS and DSS of pathological variables and BVI and LVI (Cox proportional hazard method, *n* = 256*)

Variables	Categories	Multivariable analysis RFS		Multivariable analysis DSS	
		HR (95%CI)	*p*-value	HR (95% CI)	*p*-value
Gender	Female	1		1	
	Male	0.9 (0.6–1.5)	0.92	1.1 (0.7–1.7)	0.82
Age	40–68	1		1	
	69–76	1.0 (0.7–1.6)	0.90	1.4 (0.8–2.4)	0.21
	77–88	0.6 (0.4–1.2)	0.13	1.1 (0.8–1.4)	0.71
Pathological stage	pT1 + 2	1		1	
	pT3 + 4	2.4 (1.5–3.9)	0.001	2.2 (1.3–3.7)	0.005
Nodal status	N0	1		1	
	N1–3	2.6 (1.7–3.9)	< 0.001	2.6 (1.7–4.1)	< 0.001
BVI	Negative	1		1	
	Positive	1.5 (1.0–2.3)	0.035	1.6 (1.0–2.4)	0.039
LVI	Negative	1		1	
	Positive	0.8 (0.6–1.2)	0.36	0.9 (0.6–1.3)	0.44
Surgical margins	Negative	1		1	
	Positive	1.9 (1.1–3.1)	0.016	1.8 (1.1–3.0)	0.027
Perioperative chemotherapy	No	1		1	
	Yes	0.9 (0.6–1.4)	0.65	0.9 (0.5–1.4	0.57

Abbreviations: *RFS* recurrence free survival; *DSS* disease specific survival *HR* hazard ratio; *95% CI* 95% confidence interval; *BVI* blood vessel invasion, *LVI* lymph vessel invasion. *; performed lymph node dissection

There was no interaction between BVI and LVI (RFS; *p* = 0.78, DSS; *p* = 0.73), and the impact of BVI and LVI did not change when exclusively including either of them in the model.

The 2-year observed DSS and OS for patients with BVI were 38 and 37%, compared to 73 and 69% for patients without BVI. For patients with LVI, the 2-year DSS and OS were 56 and 51%, compared to 67 and 65% for patients without LVI.

#### BVI analysis of the pN0 subgroup

30 (18%) of the patients with negative lymph nodes showed BVI, of which 14 patients experienced relapse of disease. When analysing the pN0 subgroup, BVI was significantly associated with reduced RFS in univariable analysis (HR 2.0, CI 1.1–3.7, *p* = 0.0024). For DSS, we observed a trend for increased risk for BC-related deaths in the pN0 subgroup with BVI (HR 1.9, CI 0.9–3.6, *p* = 0.060).

#### VI by standard HAS sections

VI assessed without IHC was significantly associated with poorer RFS and DSS in univariable analyses (*p* < 0.001) (Supplementary Fig. 1, additional file 1) When adjusting for basic clinicopathological variables, there was still a trend for increased risk of BC-related death for individuals with VI (HR 1.5, CI 0.9–2.2, *p* = 0.084) (Table 4).

**Table 4:** Multivariable analyses of RFS and DSS (Cox proportional hazard method) of pathological variables and vascular invasion assessed without immunohistochemistry (*n* = 256*)

Variables	Categories	Multivariable analysis RFS		Multivariable analysis DSS	
		HR (95%CI)	*p*-value	HR (95% CI)	*p*-value
Gender	Female	1			
	Male	0.9 (0.6–1.5)	0.81	1.0 (0.7–1.6)	0.90
Age	40–68	1		1	
	69–76	1.0 (0.6–1.7)	0.26	1.4 (0.8–2.2)	0.22
	77–88	0.7 (0.4–1.2)	0.20	1.2 (0.7–2.1)	0.52
Pathological stage	pT1 + 2	1		1	
	pT3 + 4	2.4 (1.4–3.9)	0.001	2.1 (1.3–3.7)	0.005
Nodal status	N0	1		1	
	N1–3	2.4 (1.6–3.7)	< 0.001	2.4 (1.6–3.8)	< 0.001
VI	Negative	1		1	
	Positive	1.3 (0.9–1.9)	0.19	1.5 (0.9–2.2)	0.084
Surgical margins	Negative	1		1	
	Positive	1.9 (1.1–3.1)	0.013	1.8 (1.1–3.0)	0.025
Perioperative chemotherapy	No	1		1	
	Yes	0.9 (0.6–1.4)	0.60	0.9 (0.5–1.4)	0.59

Abbreviations: *HR* hazard ratio; *95% CI* 95% confidence interval; *VI* vascular invasion assessed on HAS sections. *; performed lymph node dissection

## Discussion

This study showed 32% LVI and 28% BVI. To the authors´ knowledge, this is the largest study to date investigating LVI and BVI by IHC in cystectomy specimens. Studies in which morphological criteria to separate the vessel types have been applied have shown prevalences of LVI varying between 20.8–54.1%, and BVI between 4-29.8% [9–12]. A few of these studies have found BVI but not LVI to be an independent predictor for disease specific survival [9, 12]. The wide range in reported prevalences reflects the acknowledged challenge of recognizing true lymphatic and blood vessel spaces. As a novel finding, we observed 64% increased sensitivity in detecting VI when applying IHC on one selected tumor block, of which 25 cases represented BVI. Immunohistochemical markers of blood and lymphatic endothelium obviously increase the sensitivity and specifisity of the assessment of vascular spaces. Despite this, according to current recommendations, immunohistochemistry should only apply on equivocal cases [19, 24]. LVI is more frequently observed than BVI in most cancers studied. Studies of breast cancer using IHC [25, 26] have reported LVI in 25–35% of cases, and BVI in 15–16%. In endometrial, colon and cervical carcinomas, the prevalences reported have been 31, 54 and 20.4% for LVI, and 18, 41 and 11.2% for BVI, respectively [14, 27, 28]. In gastric cancer on the other hand, BVI has been shown to be more prevalent than LVI [29, 30].

Former studies [9, 10, 12, 13] have reported VI prevalences of 30–50%, based on assessment without IHC. We observed a prevalence of 31%. Known interobserver variability concurs with our finding of an interobserver agreement of 60%.

In this study, we found that BVI has a stronger association with recurrence and reduced survival than LVI. There is a paucity of studies using IHC in this field. Of note is the work of Afonso et al. [17] reporting that BVI remained an independent prognostic factor on multivariable analysis of OS in this rather small series. In several studies were IHC has not been applied, VI assessed on H&E has been found to be independently associated with decreased survival regardless of lymph node status [6, 11]. Yuk et al. [31] reported that lymphovascular invasion had a similar prognostic value as lymph node involvement in patients undergoing RC. In our study, when analyzing the pN0 subgroup, BVI showed significant association with reduced RFS in univariable analysis. For DSS, we observed a similar trend for increased risk for BC-related deaths in the pN0 subgroup with BVI, although the numbers are small and larger series are needed to confirm our observations.

Our findings might indicate that some tumors are inclined to direct hematogenous spread. This effect could explain why BVI remains an independent prognostic factor in multivariable survival analyses. Notably, studies of disseminated tumor cells from the bone marrow indicate that hematogenous spread is often an early event in tumor progression [32, 33]. Our results suggest that immunohistochemical assessment of BVI and LVI on a regular basis may increase detection rates and could contribute to selecting patients for adjuvant therapy, particularly in the pN0 group.

VI detected without IHC is considered a strong prognostic factor. However, in our study, when adjusting for basic clinicopathological variables we did not find this feature to be significant in multivariable RFS and DSS survival analyses, although we observed a trend for increased risk of BC-related death for individuals with VI detected on HAS sections. Similar results have been described in other studies [34–36].

As for tumors located in the bladder neck area, our findings are in line with previously reported associations between VI assessed by H&E and bladder neck involvement [37, 38]. However, to our knowledge, the association between bladder neck involvement and vascular invasion separated by vessel type, BVI, as demonstrated in our study has not been previously established. It has been suggested that the dense plexus of veins surrounding the bladder neck may be an easier conduit for VI [37]. Bladder neck involvement may therefore contribute to select higher risk patients for up-front radical treatment. Furthermore, our findings of significant association between micropapillary divergent differentiation and LVI are consistent with previously reported observations in the literature [39–41]. It is well known that separating true VI from lacunar spaces due to tissue artifacts is particularly difficult in micropapillary divergent cases and that detection rates increase with the use of immunohistochemistry [41].

Immunohistochemical assessment revealed a greater variation in blood vessels regarding size. In many specimens, BVI was observed in small vessels morphologically indistinguishable from small lymph vessels, or retraction lacunae in some cases. We believe that the observed higher sensitivity in detecting vessel invasion by using IHC partly can be explained by the facilitated detection of BVI, in particular, in small venules. We also noted that CD31 positivity might be scarce in blood vessels of larger size.

We recognize that our study have some limitations. The study is retrospective in design, spanning 18 years, without standard procedures of the sampling of neither the cystectomy nor lymph node specimens. There are probable changes in surgical and oncological practice as well as inherent biases in the study cohort that could have impact on outcome. In particular, the exclusion of ypT0 cases due to study design will have affected both the observed rate of neoadjuvant therapy in the cohort as well as it may explain the estimated lack of impact of neoadjuvant therapy when adjusting for this factor in the survival analyses. On the other hand is the follow up comprehensive and complete.

Of the 91 cases classified as VI positive on HAS sections in our study, IHC could not confirm this in 6 cases (7%). This could be due to misinterpretation in cases where artificial tissue shrinkage might have given the impression of vascular spaces. A contributing factor might have been the cutting of deeper sections when performing IHC. The choosing of a single section of the total cystectomy specimen to perform IHC could further imply that other areas of BVI and LVI might have been missed. Most cases of LVI visualized by D2–40 were apparently easy to interpret, although some staining in myofibroblastic cells might represent a source of misclassification towards false positive assessment, as D2–40 previously has been shown to be a marker for urinary bladder myofibroblasts [42, 43].

## Conclusion

Even though LVI was found to be associated with several aggressive tumor features, and in particular demonstrated the ability to independently predict regional lymph node metastases, BVI indicated a poorer prognosis. Our findings concur with the general view that BVI is associated with more widespread metastases to distant organs. In survival analyses, BVI was significantly associated with shorter RFS and DSS whereas LVI was not. In conclusion, our study has shown that in invasive BC, lymphatic involvement is more frequent than BVI, but blood vessel involvement is a stronger prognostic factor, although more studies need to confirm our observations. Furthermore, associations between blood and lymph vessel invasion and BC molecular subtypes ought to be clarified in future studies. The importance of distinguishing blood vessel invasion from lymphatic invasion in the pathology report is implied.

## Supplementary Information

**Additional file 1: Table S1.** Associations between LVI and BVI assessed by IHC. **Table S2.** Associations between VI assessed by HAS and by IHC. **Table S3.** Associations between lymph node metastases and various clinicopathological variables (logistic regression). **Table S4.** Associations between higher pathological stages and various clinicopathological variables (logistic regression). **Fig. S1.** Kaplan Meier curves RFS (A) and DSS (B), VI assessed without IHC. **Fig. S2.** Kaplan Meier curves RFS (A) and DSS (B), different categories of VI assessed by IHC. Footnotes: Survival curves are estimated by the Kaplan Meier method using the log-rank test for differences between subgroups. For each category, number of events/total number of cases are given.

## Data Availability

All data generated or analysed during this study are included in this published article and its supplementary information files.

## Abbreviations

VI: Lymphovascular invasion
LVI: Lymph vessel invasion
BVI: Blood vessel invasion
IHC: Immunhistochemistry
HAS: Hematoxylin-azophloxine-saffron
RC: Radical cystectomy
RFS: Recurrence free survival
DSS: Disease specific survival
BC: Bladder cancer
OS: Overall survival
OR: Odds ratio
CI: Confidence interval
HR: Hazard Ratio
IQR: Interquartile range
