# Supplementary material for: Tumor cell invasion in blood vessels assessed by immunohistochemistry is related to decreased survival in patients with bladder cancer treated with radical cystectomy
Source: Diagn Pathol. 2021 Nov 22;16:109. doi: 10.1186/s13000-021-01171-7 (PMC8609845; doi:10.1186/s13000-021-01171-7)

| **Supplementary Table 1** Associations between LVI and BVI assessed by IHC. | | | | | | | | | | | | | |
| --- | --- | --- | --- | --- | --- | --- | --- | --- | --- | --- | --- | --- | --- |
|  | | | BVI | | | | | | | | | | |
|  | | | Negative (n, %) | | Positive (n, %) | | | | | | OR (95% CI) | | p value |
| LVI | | |  | |  | | | | | |  | |  |
| negative | | | 142 (67) | | 58 (72) | | | | | |  | |  |
| positive | | | 69 (33) | | 23 (28) | | | | | | 0.8 (0.5-1.4) | | 0.48 |
| P value was obtained using Pearson´s χ² test. | | | | |  | |  | | |  | | |  |
|  | | | | |  | |  | | |  | | |  |
| **Supplementary Table 2** Associations between VI assessed by HAS, and LVI and BVI assessed by IHC. | | | | | | | | | | | | | |
|  | | VI assessed by HAS | | | | | | | | | | | |
|  | | Negative (n, %) | | Positive (n, %) | | | | | OR (95% CI) | | | p value | |
| BVI  Negative | 176 (88) | | | 35 (38) | | | | |  | | |  | |
| positive | 25 (12) | | | 56 (62) | | | | | 11.3 (6.2-20.4) | | | <0.001 | |
|  |  | | |  | | | | |  | | |  | |
| LVI |  | | |  | | | | |  | | |  | |
| negative | 153 (76) | | | 47 (52) | | | | |  | | |  | |
| positive | 48 (24) | | | 44 (48) | | | | | 2.9 (1.8-5.0) | | | <0.001 | |
| P values were obtained using Pearson´s χ² test. | | | |  | |  | |  | | | |  | |

| **Supplementary Table 3:** Multivariable analyses of associations between regional lymph node metastasis and various clinicopathological variables including BVI and LVI (logistic regression) (n=240) | | | | | | | | | | | | | |
| --- | --- | --- | --- | --- | --- | --- | --- | --- | --- | --- | --- | --- | --- |
| Variables | Categories | | OR (95% CI) | | | | | | P value | | | | |
|  |  | | | |  | | |  | | |  | |  |
| Gender | | Male | | | | 1 | | | |  | |  |  |
|  | | Female | | 1.9 (0.9-4.3) | | | | | | 0.10 | |  |  |
|  | |  | |  | | | | | |  | |  |  |
| Age | | 40-68 | | 1 | | | | | |  | |  |  |
|  | | 69-76  77-88 | | 1.2 (0.5-2.7)  0.9 (0.4-2.3) | | | | | | 0.64  0.99 | |  |  |
|  | |  | |  | | | | | |  | |  |  |
| Pathological stage | | pT1+2 | | 1 | | | | | |  | |  |  |
|  | | pT3+4 | | 6.2 (2.9-13.4) | | | | | | <0.001 | |  |  |
|  | |  | |  | | | | | |  | |  |  |
| Micropapillary div. diff. | | Negative | | 1 | | | | | |  | |  |  |
|  | | Positive | | 4.5 (1.5-14.2) | | | | | | 0.009 | |  |  |
|  | |  | |  | | | | | |  | |  |  |
| BVI | | Negative | | 1 | | | | | |  | |  |  |
|  | | Positive | | 2.4 (1.2—4.8) | | | | | | 0.018 | |  |  |
|  | |  | |  | | | | | |  | |  |  |
| LVI | | Negative  Positive | | 1  2.7 (1.4-5.2) | | | | | | 0.003 | |  |  |
|  | |  | |  | | | | | |  | |  |  |
| Neoadjuvant chemotherapy | | No  Yes | | 1  0.6 (0.2-1.5) | | | | | | 0.25 | |  |  |
|  |  | |  | | | |  | |  | | | |  |
| Abbreviations: OR, odds ratio, 95% CI; 95% confidence interval. | | | | | | | | | | | | | |

| **Supplementary Table 4:** Multivariable analyses of associations between higher pathological stages and various clinicopathological variables including BVI and LVI (logistic regression) (n=240) | | | | | | | | | | | | |
| --- | --- | --- | --- | --- | --- | --- | --- | --- | --- | --- | --- | --- |
| Variables | Categories | | OR (95% CI) | | | | | P value | | | | |
|  |  | | |  | | |  | | |  | |  |
| Gender | | Male | | | 1 | | | |  | |  |  |
|  | | Female | | | 1.4 (0.7-2.9) | | | | 0.31 | |  |  |
|  | |  | | |  | | | |  | |  |  |
| Age | | 40-68 | | | 1 | | | |  | |  |  |
|  | | 69-76  77-88 | | | 0.7 (0.3-1.4)  1.2 (0.5-2.6) | | | | 0.33  0.60 | |  |  |
|  | |  | | |  | | | |  | |  |  |
| Lymph node metastasis | | pN0 | | | 1 | | | |  | |  |  |
|  | | pN1-3 | | | 6.2 (2.9-13.5) | | | | <0.001 | |  |  |
|  | |  | | |  | | | |  | |  |  |
| Micropapillary div. diff. | | Negative | | | 1 | | | |  | |  |  |
|  | | Positive | | | 0.4 (0.1-1.3) | | | | 0.13 | |  |  |
|  | |  | | |  | | | |  | |  |  |
| BVI | | Negative | | | 1 | | | |  | |  |  |
|  | | Positive | | | 3.9 (1.8-8.8) | | | | 0.001 | |  |  |
|  | |  | | |  | | | |  | |  |  |
| LVI | | Negative  Positive | | | 1  1.5 (0.8-2.9) | | | | 0.23 | |  |  |
|  | |  | | |  | | | |  | |  |  |
| Neoadjuvant chemotherapy | | No  Yes | | | 1  1.4 (0.6-2.9) | | | | 0.42 | |  |  |
|  |  | |  | | |  | |  | | | |  |
| Abbreviations: OR, odds ratio, 95% CI; 95% confidence interval. | | | | | | | | | | | | |

**Supplementary Figure 1**.

A B


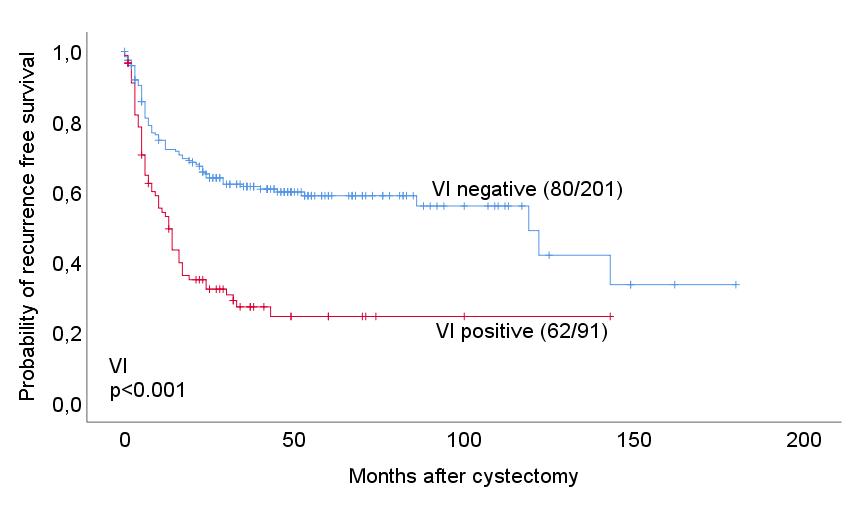

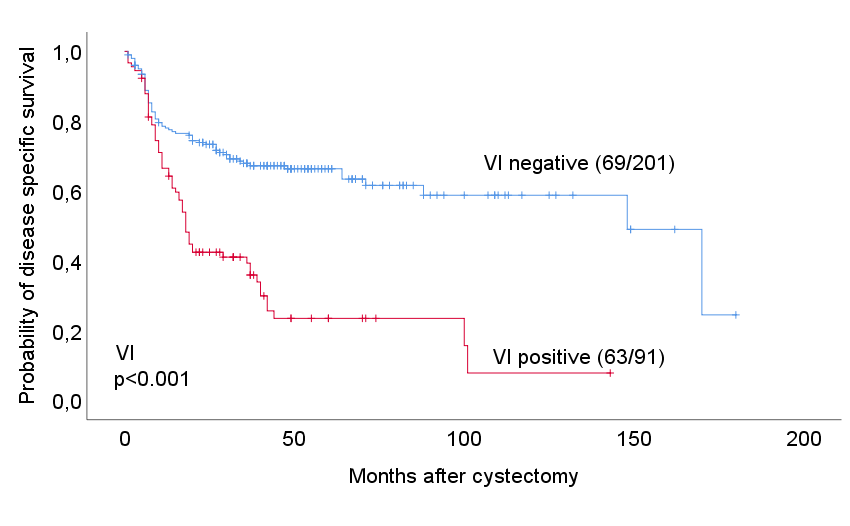


**Supplementary Figure 2**.

A B
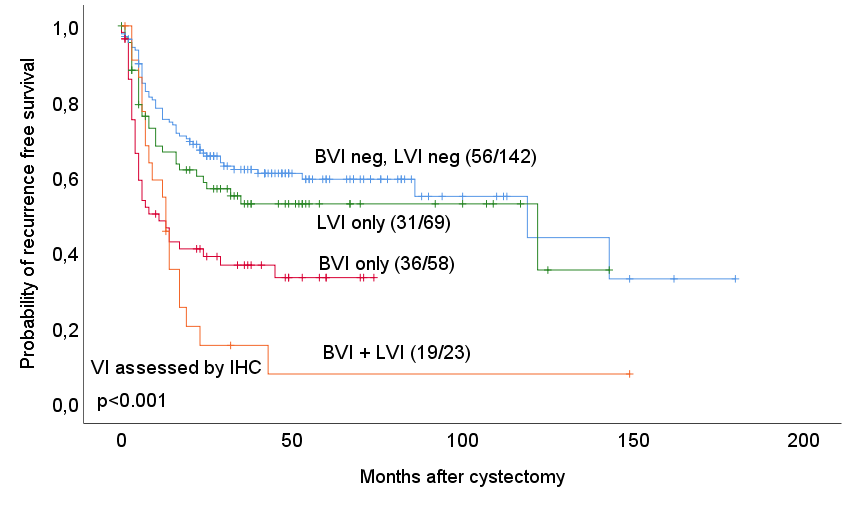

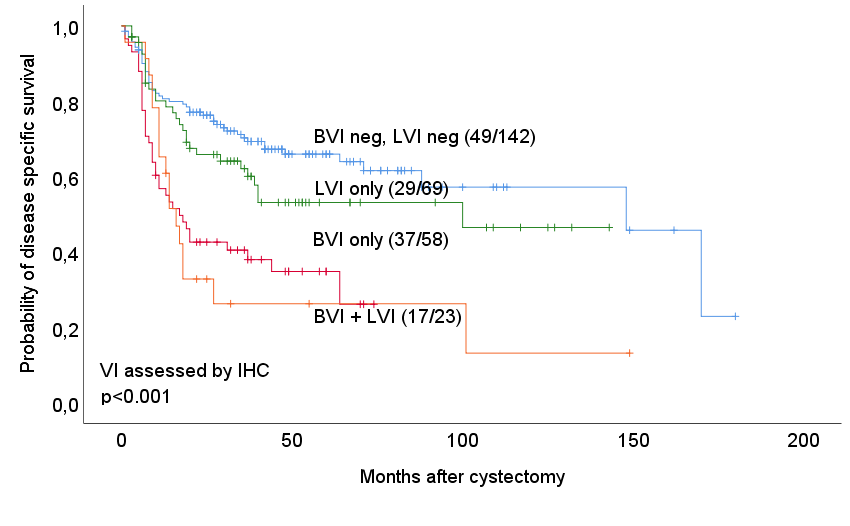

Supplement: Supplementary file 1 — Additional file 1: Table S1. Associations between LVI and BVI assessed by IHC. Table S2. Associations between VI assessed by HAS and by IHC. Table S3. Associations between lymph node metastases and various clinicopathological variables (logistic regression). Table S4. Associations between higher pathological stages and various clinicopathological variables (logistic regression). Fig. S1. Kaplan Meier curves RFS (A) and DSS (B), VI assessed without IHC. Fig. S2. Kaplan Meier curves RFS (A) and DSS (B), different categories of VI assessed by IHC. Footnotes: Survival curves are estimated by the Kaplan Meier method using the log-rank test for differences between subgroups. For each category, number of events/total number of cases are given. [file 13000_2021_1171_MOESM1_ESM.docx]
